# Decoding the Generational Digital Divide: Profiles and Predictors of Grandparents’ Attitudes Toward Young Children’s Technology Use

**DOI:** 10.3390/bs16050832

**Published:** 2026-05-21

**Authors:** Wenwei Luo, Huihua He, Ilene R. Berson, Michael J. Berson, Zhiying Wang

**Affiliations:** 1 Shanghai Institute of Early Childhood Education, Shanghai Normal University, Shanghai 200234, China; wenweiluo@shnu.edu.cn; 2 Department of Teaching and Learning, University of South Florida, Tampa, FL 33620, USA; iberson@usf.edu (I.R.B.); berson@usf.edu (M.J.B.); 3 School of Education, Shanghai Normal University, Shanghai 200234, China

**Keywords:** grandparents, grandchildren, attitudes, technology use

## Abstract

In today’s digital age, child-rearing presents unique challenges that extend across generations, impacting both parenting and grandparenting. This study investigated patterns of grandparents’ attitudes toward their grandchildren’s technology use and identified key predictors of these patterns. Utilizing latent profile analysis (LPA) with a sample of 712 grandparents from Shanghai, China, the research identified four distinct attitudinal profiles: positively supportive, cautiously observant, low-involvement reserved, highly concerned and restrictive. Multinomial logistic regression analyses revealed that the age of the grandparent, sibling status, and the frequency of communication between grandparents and parents were significant predictors of profile membership. The findings indicate that grandparents’ attitudes toward their grandchildren’s technology use reflect a combination of acceptance and concern. This study underscores the need for further research and educational supports to help grandparents develop perspectives informed by an evidence base, thereby strengthening guidance strategies for young children’s digital engagement.

## 1. Introduction

Infants, toddlers, and preschoolers today are raised in environments saturated with digital technologies, including computers, televisions, mobile phones, and tablets (Byrne et al., 2021; Dong et al., 2022; Zhao et al., 2018). The increasing amount of time young children spend using these devices presents new challenges for caregiving. While digital parenting—encompassing parents’ perceptions, beliefs, and mediation strategies regarding children’s technology use—has been widely examined (Barkin et al., 2006; Sun et al., 2021; K. V. Tan & Zhooriyati, 2021), there is limited research on grandparents’ attitudes toward children’s technology use. This gap highlights the importance of investigating grandparents’ perspectives as a crucial and emerging area of inquiry in the digital age.

From a psychological perspective, attitudes refer to the degree of favorability or unfavourability an individual holds toward a particular object or situation, shaped by cognitive, emotional, and behavioral components (Edison & Geissler, 2003; Hernández-Ramos et al., 2014). In caregiving contexts, attitudes toward children’s technology use are influenced by beliefs about the role and value of digital technologies in children’s development (Aesaert & van Braak, 2015). Prior research has largely focused on parental attitudes and their influence on children’s technology-related behaviors and dispositions (Türel & Gür, 2019; D. Wu et al., 2020). However, grandparents also play an active role in shaping children’s everyday experiences, and their attitudes may reinforce, challenge, or complement parental approaches to technology use.

Despite this, existing research remains predominantly centered on parents, resulting in a limited understanding of grandparents’ perspectives (Gür & Türel, 2022; K. V. Tan & Zhooriyati, 2021). Addressing this gap is essential for advancing the concept of digital grandparenting and for developing a more comprehensive understanding of family influences on young children’s technology use. Accordingly, this study aims to validate an assessment instrument and to identify distinct patterns and key predictors of grandparents’ attitudes toward their grandchildren’s technology use.

### 1.1. Grandparenting and Children’s Technology Use in China

Across the world, grandparents serve as a critical childcare safety net, regularly providing care for their grandchildren on a weekly basis (Di Gessa et al., 2020; Floridi, 2022; Hamilton & Suthersan, 2021; National Association of Child Care Resource & Referral Agencies [NACCRRA], 2008). Grandparental involvement in childcare is particularly prevalent in China, where intergenerational caregiving has become a prominent feature of family life and early childhood care arrangements (Lu et al., 2020; Settles et al., 2009). A national survey in China indicates that 73.29% of grandparents provide intergenerational care for their grandchildren (Huang et al., 2016). In comparison, approximately 30% of families with children under age five in the United Kingdom receive grandparental support (Gray, 2005). In the United States, nearly 60% of grandparents have either provided or are currently providing childcare for their grandchildren, highlighting their central role in family caregiving (National Association of Child Care Resource & Referral Agencies [NACCRRA], 2008). Previous research has examined the sociocultural and economic factors underlying the widespread reliance on grandparents for childcare in China (Bao, 2019; Zheng, 2019). In many Chinese families, grandparents are not only practical caregivers but also active participants in children’s everyday learning and development.

As digital technologies become increasingly embedded in young children’s everyday lives, grandparents are also becoming important mediators of children’s digital experiences. Existing studies consistently suggest that children frequently engage in screen-based and digital activities while under grandparental care, including television viewing, smartphone use, and other technology-related activities (Dunifon et al., 2018; Elias et al., 2019; Gou & Dezuanni, 2018). In China, children cared for primarily by grandparents have been reported to spend more time watching television and using smartphones than children cared for mainly by their parents (Gou & Dezuanni, 2018). In Shanghai, one of the most economically developed regions in China, children’s engagement with digital technology is particularly extensive. Zhao et al. (2018) reported that the average daily screen time of children aged 3–4 years substantially exceeded the limits recommended by the American Academy of Pediatrics (Hill et al., 2016).

Despite grandparents’ substantial involvement in children’s digital experiences, most existing research on caregivers’ attitudes toward children’s technology use has focused primarily on parents (Anastasiades et al., 2008; Bartau-Rojas et al., 2018; Domingues-Montanari, 2017; Gür & Türel, 2022). Comparatively little attention has been paid to grandparents, even though they may differ from younger parents in digital literacy, media experiences, caregiving practices, and perceptions of technology-related risks and benefits. As a result, grandparents’ attitudes toward children’s technology use may reflect distinctive intergenerational patterns that cannot be fully understood through parent-based research alone. This study therefore addresses an important gap by examining the patterns and predictors of grandparents’ attitudes toward their grandchildren’s technology use.

### 1.2. Caregivers’ Attitudes Toward Children’s Technology Use: Comparing Parents and Grandparents

Research on caregivers’ attitudes toward children’s use of digital technologies has predominantly focused on parents. Existing studies generally conceptualize caregivers’ attitudes as involving interrelated cognitive, affective, and behavioral dimensions, including beliefs about the educational value of technology, concerns about developmental and safety risks, and intentions regarding supervision and regulation of children’s technology use. These attitudes are reflected in decisions about how much freedom versus control to allow, how to balance educational benefits against potential risks, and the degree of parental oversight required. For example, parents may support the use of digital tools for learning while simultaneously imposing limits to address concerns about screen time or online safety (Oladejo et al., 2016; Szpunar et al., 2022; B. Q. Tan et al., 2019). In this sense, caregivers’ attitudes shape not only how children access digital technologies but also how digital experiences are integrated into everyday family life (Scott, 2022).

Prior research (Türel & Gür, 2019) identifies three key dimensions of parental attitudes toward children’s technology use: educational value, control and limitations, and negative effects. These dimensions encompass the perceived educational potential of technology, concerns about risks, and the use of restrictions to mitigate those risks (Gür & Türel, 2022). Given that grandparents also play a caregiving role, it is important to consider whether this framework can be extended to capture their attitudes. Accordingly, this study examines grandparents’ perspectives by focusing on their views of the educational benefits of technology, its impact on child development, and their role in mediating its use.

Research on parents’ attitudes toward children’s technology use reflects a lack of consensus. Some parents hold positive views, believing that technology can support the development of skills such as critical thinking, social–emotional competencies, literacy, numeracy, and technological literacy (Anastasiades et al., 2008; Bartau-Rojas et al., 2018; Gou & Dezuanni, 2018; Jiang & Monk, 2016; Soykan, 2015). In particular, Chinese parents are more likely to emphasize the educational functions of technology. For example, Dong et al. (2022) found that while Chinese parents prioritize educational benefits, parents in European contexts tend to place greater emphasis on entertainment. Similarly, studies suggest that grandparents also value the educational potential of technology for children (Jiang & Monk, 2016).

At the same time, some parents express concerns about the potential negative effects of digital technology, including sleep disruption, vision problems, obesity, digital dependency, and online safety risks (Domingues-Montanari, 2017; Jiang & Monk, 2016; Li et al., 2022; Sothern, 2004). These concerns can be broadly categorized as content, exposure, and behavioral risks (Smahelova et al., 2017). Although fewer studies have examined grandparents’ perspectives, existing research indicates that they share similar concerns, particularly regarding the impact of excessive screen time on children’s physical and social–emotional development (Xie et al., 2018).

Moreover, caregivers’ attitudes toward children’s technology use are closely linked to the mediation strategies they employ (Connell et al., 2015; Nikken & Schols, 2015; Sun et al., 2021; K. V. Tan & Zhooriyati, 2021). For example, parents who perceive developmental benefits are more likely to support technology use and apply fewer restrictions (K. V. Tan & Zhooriyati, 2021). Parental mediation—defined as the strategies used to guide and regulate children’s technology use (Clark, 2011)—has been shown to shape children’s digital experiences (Rodríguez-de-Dios et al., 2018; Vekiri & Chronaki, 2008). These strategies are typically categorized as active mediation, restrictive mediation, monitoring, and co-use (Dong et al., 2022; Livingstone & Helsper, 2008). Nikken and Schols (2015) found that parents with more positive attitudes tend to use monitoring, co-use, and active mediation, whereas those with more negative attitudes rely more heavily on restrictive approaches.

One study categorized grandparents into three groups based on these mediation strategies: highly involved mediators, unrestricted mediators, and less involved mediators (Nimrod et al., 2022). The findings indicated that most grandparents were highly involved, engaging in multiple mediation practices. This suggests that grandparents’ mediation strategies, like those of parents, may be shaped by their attitudes toward children’s technology use.

Although research on caregivers’ attitudes toward children’s digital technology use has largely centered on parents, studies examining grandparents’ perspectives remain limited. Given grandparents’ involvement in children’s digital activities (Dunifon et al., 2018; Nimrod et al., 2022), it is important to examine how they perceive the role and value of digital technology in their grandchildren’s lives.

### 1.3. Predictors of Grandparents’ Attitudes

Existing research suggests that grandparents’ attitudes toward children’s digital technology use may be shaped by a combination of demographic, caregiving, and sociocultural factors. Prior studies consistently indicate that caregivers who are younger, more highly educated, and more familiar with digital technologies tend to hold more positive attitudes toward technology use and demonstrate greater openness toward children’s digital engagement (Edison & Geissler, 2003; Gou & Dezuanni, 2018). In addition, caregiving contexts may also influence technology-related attitudes and mediation practices. For example, grandparents caring for multiple grandchildren may permit greater technology use because supervising several children simultaneously can be more challenging (Gou & Dezuanni, 2018; Nimrod et al., 2022). Similarly, intergenerational communication and guidance from parents may shape how grandparents regulate children’s technology use (Nimrod et al., 2020).

Focusing specifically on grandparents, Xie et al. (2018) reported that Latino grandfathers were more likely than grandmothers to hold favorable attitudes toward their grandchildren’s technology use, often expressing admiration for their grandchildren’s proficiency with digital devices. In addition, Gou and Dezuanni (2018) found that grandparents may permit greater technology use among grandchildren in families with multiple children, likely due to the challenges of supervising more than one child. Similarly, Nimrod et al. (2022) observed that grandparents who cared for multiple grandchildren were less involved in mediating technology use compared to those who cared for only one child. Moreover, the extent of parenting guidance grandparents receive from children’s parents may also shape their attitudes. For instance, Nimrod et al. (2020) found that grandparents who received more guidance from younger parents were more likely to adopt restrictive mediation strategies.

Cultural perspectives further contribute to shaping attitudes toward children’s technology use. Jiang and Monk (2016) suggested that the attitudes of Chinese–Australian parents and grandparents are influenced by Confucian traditions, which emphasize the importance of academic success. This cultural orientation may encourage the use of technology to support both formal and informal learning. In addition, the concept of lifelong learning is particularly relevant for older adults in a digital society, as continued learning can enhance digital literacy and support intergenerational connections (Patrício & Osório, 2011). Accordingly, grandparents’ engagement in ongoing learning may also shape their attitudes toward their grandchildren’s use of digital technology.

To anchor the present study in established theory, we draw on the Technology Acceptance Model (TAM; Davis, 1989; Granić & Marangunić, 2019) and complement it with intergenerational digital-divide theory (Bates & Taylor, 2013; Friemel, 2016). TAM proposes that perceived usefulness and perceived ease of use jointly shape individuals’ behavioral intentions and use of technology, and educational-technology research has further extended TAM to incorporate perceived risk (Granić & Marangunić, 2019). Read through this lens, the three dimensions of caregiver attitudes already identified by Türel and Gür (2019), namely educational value, control and limitations, and negative effects, correspond respectively to perceived usefulness for the child, perceived behavioral controllability and intention to regulate use, and perceived risk. This conceptual mapping motivates our use of these three dimensions as the basis for measuring grandparental attitudes in the present study; the specific instrument we adapt for that purpose is described in the Section 2.

Intergenerational digital-divide theory situates grandparents as a digitally transitional cohort whose digital access, skills, and confidence often differ from those of younger generations (Friemel, 2016). These intergenerational differences may contribute to substantial variation in how grandparents perceive the educational value, risks, and appropriate regulation of children’s technology use. Rather than falling along a simple positive–negative continuum, grandparents’ attitudes may therefore reflect heterogeneous combinations of perceived benefits, concerns, and regulatory tendencies. Such heterogeneity is also consistent with previous research identifying differentiated mediation patterns among grandparents (Nimrod et al., 2022). Accordingly, a person-centered approach such as latent profile analysis (LPA) is particularly suitable for identifying distinct attitudinal profiles among grandparents and examining how demographic and caregiving contextual factors predict profile membership.

### 1.4. The Current Study

With the rapid development of China’s economy, society, and technology, digital devices such as computers, tablets, smartphones, point-and-shoot pens, and digital early-learning machines have become integral to the daily lives of young children. A study in central China found that digital adoption levels in families with young children are approaching those observed in European and North American contexts (Dong et al., 2022). As a result, young children in China have ample opportunities to engage with a wide range of technological activities within their homes. In Shanghai, one of the most developed regions in China, children’s engagement with technology is particularly high. A survey conducted by the Shanghai Children’s Medical Centre reported that the average screen time for 3–4-year-olds is 2 h and 48 min per day (Zhao et al., 2018), which substantially exceeds the limits recommended by the American Academy of Pediatrics for children aged 2–5 years (Hill et al., 2016).

Although previous research has extensively examined parents’ attitudes toward children’s technology use, grandparents remain comparatively underexplored despite their substantial caregiving role in Chinese three-generation households. Importantly, grandparents differ from younger parents in digital literacy, caregiving experiences, intergenerational positioning, and familiarity with digital technologies. Consequently, findings derived from parent samples cannot simply be generalized to grandparents. Given grandparents’ significant involvement in young children’s everyday digital experiences, understanding their attitudes toward technology use is both theoretically and practically important.

Existing studies have primarily adopted variable-centered approaches and have generally conceptualized caregivers’ attitudes along a positive–negative continuum. However, the integration of the Technology Acceptance Model and intergenerational digital-divide theory suggests that grandparents’ attitudes may instead reflect heterogeneous configurations of perceived educational value, perceived risk, and regulatory inclination. In addition, prior studies on parental and grandparental mediation have identified differentiated caregiver patterns rather than uniform attitudinal tendencies (Nimrod et al., 2022; D. Wu et al., 2020). Therefore, a person-centered approach such as latent profile analysis (LPA) is particularly appropriate for identifying distinct attitudinal patterns among grandparents.

Furthermore, although the three-dimensional structure of caregiver attitudes toward children’s technology use—educational value, control and limitations, and negative effects—has been validated in parental research (Türel & Gür, 2019), no grandparent-specific instrument has yet been validated and applied within a person-centered analytical framework. To address these gaps, the present study aims to validate a grandparent-adapted instrument, identify distinct profiles of grandparents’ attitudes toward children’s technology use, and examine demographic and caregiving contextual predictors of profile membership. Accordingly, the study is guided by the following research questions:

1. What are the psychometric properties of the Grandparents’ Attitudes Toward Children’s Use of Technology (GACU-T) scale?
2. What distinct profiles characterize grandparents’ attitudes toward children’s technology use?
3. What personal characteristics of grandparents and grandchildren, along with caregiving contextual factors, predict profile membership?

Based on prior parental research and the theoretical framework outlined above, the following hypotheses were proposed:

**H1.**  *The three-factor structure of caregiver attitudes—educational value, control and limitations, and negative effects—identified in parental research will also be supported among grandparents.*

**H2.**  *Grandparents’ attitudes toward children’s technology use will form substantively distinct latent profiles rather than vary along a single positive–negative continuum.*

**H3.**  *Grandparent age, education, family structure, and the frequency of parent–grandparent communication will be associated with profile membership.*

## 2. Methods

### 2.1. Participants

Participants were recruited while attending a grandparenting seminar organized by a community in one district of Shanghai. In this study, electronic questionnaires were distributed to all grandparents of children aged 0–6 years who attended the seminar, resulting in 712 valid responses. All participants were fully informed about the study’s purpose and procedures.

Table 1 presents the demographic characteristics of the participating grandparents. Among the respondents, 339 (47.61%) were paternal grandmothers, followed by maternal grandmothers (25.98%), paternal grandfathers (20.23%), and maternal grandfathers (6.18%). Most participants (61.66%) were younger than 60 years. Educational attainment was generally low, with only 8.57% holding a college or university degree. In addition, 77.53% of participants were retired or unemployed.

Most grandchildren (95.51%) were between 36 and 72 months of age. Of the grandchildren, 55.20% were boys and 44.80% were girls. Approximately half were only children (50.42%), while 49.58% had one or more siblings.

### 2.2. Measures

#### 2.2.1. Grandparents’ Attitudes Toward Children’s Use of Technology (GACU-T)

The Parents’ Attitudes Toward Children’s Use of Technology (PACU-T) Scale, originally developed by Türel and Gür (2019), was adapted in the current study as the Grandparents’ Attitudes Toward Children’s Use of Technology (GACU-T) Scale to examine grandparents’ attitudes toward children’s technology use. The GACU-T scale includes three dimensions: “Educational Value” (EV, 7 items), “Control and Limitations” (CL, 6 items), and “Negative Effects” (NE, 5 items). All 18 items are rated on a five-point Likert scale ranging from 1 (strongly disagree) to 5 (strongly agree). Previous studies have confirmed the scale’s reliability and validity (Gür & Türel, 2022; Türel & Gür, 2019).

The research team contacted the original developers of the PACU-T scale and obtained approval to use and adapt it for the current study. The scale was translated into Chinese and then back-translated into English by two university professors with doctoral degrees from the United States and a doctoral student in preschool education. Due to the PACU-T scale’s strong psychometric properties, the GACU-T scale retained the original structure and all items from the PACU-T scale. However, the wording was modified to better fit the grandparenting context in China. For example, “my child” was replaced with “my grandchild” in all items, and the vague term “technology” was clarified as “devices such as computers, tablets, and mobile phones” to ensure that grandparents clearly understood the items. An example of this adaptation is the item “I monitor my child’s use of technology,” which was revised to “I monitor my grandchild’s use of devices such as computers, tablets, and mobile phones.”

To ensure the scale’s appropriateness for the local context, two professors in preschool education, two doctoral students in preschool education, and two kindergarten directors reviewed the GACU-T scale. Following their review, the experts unanimously agreed on the scale’s suitability for use in this study.

#### 2.2.2. Grandparent and Child Demographic Factors

Grandparents’ identity, age, education level, and employment status were included as covariates (see Table 1). In addition, grandchildren’s age and gender were included to account for their potential influence on grandparents’ attitudes toward technology use. Caregiving contextual factors were also considered, including the number of children, co-residence with grandchildren, and the frequency of communication between grandparents and parents. Two items assessed these contextual factors: “Do you live with your grandchildren? (1 = yes, 2 = no)” and “How often do you communicate with your grandchildren’s parents about parenting? (1 = never, 2 = rarely, 3 = occasionally, 4 = frequently).”

These covariates were selected because prior research has linked caregiver age, education, family structure, and intra-family communication to caregivers’ technology-related attitudes and mediation strategies (Jiang & Monk, 2016; Nimrod et al., 2022; Sun et al., 2021).

### 2.3. Procedures

The study was reviewed and approved by the Academic Ethics Committee of Shanghai Normal University (IRB#2022054). The research team contacted the community administrative office to explain the study’s purpose and procedures and obtained informed consent from grandparents participating in a grandparenting seminar program. Questionnaires were distributed and collected via Wenjuanxing (www.wjx.cn), a widely used online survey platform in China. A total of 712 valid questionnaires were completed and submitted by participating grandparents.

### 2.4. Data Analysis

Data were analyzed using SPSS Statistics 31.0, AMOS 24.0, and R software 4.5.2. To address the first research question, we conducted item analysis, exploratory factor analysis (EFA), reliability analysis, and confirmatory factor analysis (CFA) to assess the reliability and validity of the GACU-T Scale.

For the second and third research questions, a three-step latent profile analysis (LPA) was performed to identify latent profiles of grandparents’ attitudes toward their grandchildren’s technology use and to examine demographic and caregiving contextual predictors of profile membership (Asparouhov & Muthén, 2014). In step one, LPA was conducted to identify latent classes without including covariates. In step two, participants were assigned to their most likely profile based on classification probabilities. In step three, latent class membership was used in a subsequent model to examine its associations with covariates (e.g., demographic variables such as child gender and family income).

The optimal number of profiles was determined using multiple fit indices, including the Akaike Information Criterion (AIC), Bayesian Information Criterion (BIC), sample-size adjusted BIC (aBIC), the Bootstrapped Likelihood Ratio Test (BLRT), and entropy (Nylund et al., 2007). Model selection also considered the interpretability and theoretical relevance of the profiles, as well as the proportion of participants in each profile.

## 3. Results

### 3.1. Psychometric Properties of GACU-T

#### 3.1.1. Item Analysis

Item analysis was conducted to assess the discriminative power of the 18 items. In the first step, total scores were calculated, and participants were divided into high- and low-score groups based on the top 27% and bottom 27% of the total distribution (Kelley, 1939). Independent-samples t-tests were then conducted to compare the item scores of the two groups. The results showed that all items significantly differentiated between the high- and low-score groups (*p* < 0.001), with t values ranging from 4.64 to 10.43 (M. L. Wu, 2010). In the second step, corrected item–total correlations were examined to assess the relationship between each item and the overall scale score. The coefficients ranged from 0.31 to 0.58, all exceeding the recommended minimum value of 0.30 (Fu et al., 2009).

In the third step, reliability analysis was performed to determine whether deleting individual items would improve the internal consistency of each dimension. Following M. L. Wu (2010), items were considered for removal if deleting them increased the reliability coefficient of the corresponding dimension. The results showed that removing EV2 and CL6 improved the internal consistency of their respective dimensions, i.e., the educational value dimension’s α increased from 0.886 to 0.908, and the control and limitations dimension’s α increased from 0.882 to 0.903. Although all items met the minimum criteria in the previous two analyses, EV2 and CL6 performed relatively weaker compared to the other items. Therefore, based on the combined evidence from the three item analysis procedures, these two items were removed. As a result, the final GACU-T scale retained 16 items.

The deletion of EV2 and CL6 was supported not only by these statistical considerations but also by content-validity evidence. Specifically, post-hoc review by an expert panel of two early-childhood-education professors and two kindergarten directors indicated that the original wording of EV2 and CL6 reflected a parental rather than a grandparental caregiving framing (Holden & Edwards, 1989). The decision to remove the two items therefore rests on converging psychometric and content-validity evidence.

#### 3.1.2. Exploratory Factor Analysis

Exploratory factor analysis (EFA) was conducted using SPSS Statistics 31.0 on a randomly selected half of the sample (*n* = 356) to examine the factor structure of the GACU-T scale. The Kaiser–Meyer–Olkin (KMO) measure of sampling adequacy and Bartlett’s test of sphericity indicated that the data were suitable for factor analysis (KMO = 0.861, Bartlett’s test: *χ*^2^ = 3691.957, *df* = 120, *p* < 0.001).

Principal component analysis with Kaiser’s normalized maximum variance rotation revealed three factors with eigenvalues greater than one, which together explained 70.454% of the total variance. The eigenvalues for the three factors were 5.092, 3.557, and 2.623, respectively.

Factor loadings for all items on the scale ranged from 0.724 to 0.887, exceeding the recommended threshold of 0.450, while communalities ranged from 0.538 to 0.817, all above the minimum criterion of 0.200 (see Table 2). These results indicate that all items met acceptable standards, and no items were removed. Following rotation, the three-factor structure and item composition were consistent with the original GACU-T scale.

Furthermore, the correspondence between the items and the three constructs was fully consistent with the GACU-T scale developed by Türel and Gür (2019). The first factor, which explained 31.827% of the variance, comprised 6 items from the original “Educational Value” construct. The second factor, explaining 22.234% of the variance, included 5 items from the original “Control and Limitations” construct. The third factor, accounting for 16.393% of the variance, consisted of 5 items from the original “Negative Effects” construct. Consequently, the three constructs were retained as “Educational Value (EV)”, “Control and Limitations (CL)”, and “Negative Effects (NE)” for the three constructs of the GACU-T scale.

#### 3.1.3. Reliability Analysis

Cronbach’s α analyses were conducted to assess the internal consistency of the GACU-T scale. As shown in Table 3, the Cronbach’s α for the total scale was 0.754, and the α values for the three factors were 0.908, 0.903, and 0.880, respectively, indicating strong internal consistency across the scale.

#### 3.1.4. Confirmatory Factor Analysis (CFA)

CFA was conducted using AMOS 24.0 software to validate the three-factor structure suggested by the EFA results, using the second half of the sample (*n* = 356). Standardized factor loadings for the 16 items ranged from 0.602 to 0.880, with all loadings statistically significant (*p* < 0.001).

Model fit indices indicated an acceptable fit for the three-factor model: *χ*^2^ = 354.335, *df* = 101, *χ*^2^*/df* = 3.508, *p* < 0.001, RMSEA = 0.084, GFI = 0.884, CFI = 0.923, TLI = 0.909, and IFI = 0.924. Typically, a *χ*^2^/*df* value below 5 suggests an acceptable fit, and RMSEA values below 0.08 are considered reasonable, with values between 0.08 and 0.10 indicating an acceptable fit (Gemmel et al., 2008; L. Hu & Bentler, 1999; Nonnemaker & Homsi, 2007). Moreover, IFI, TLI, and CFI values above 0.90 indicate a good model fit (Müller et al., 2013).

**Figure 1:**
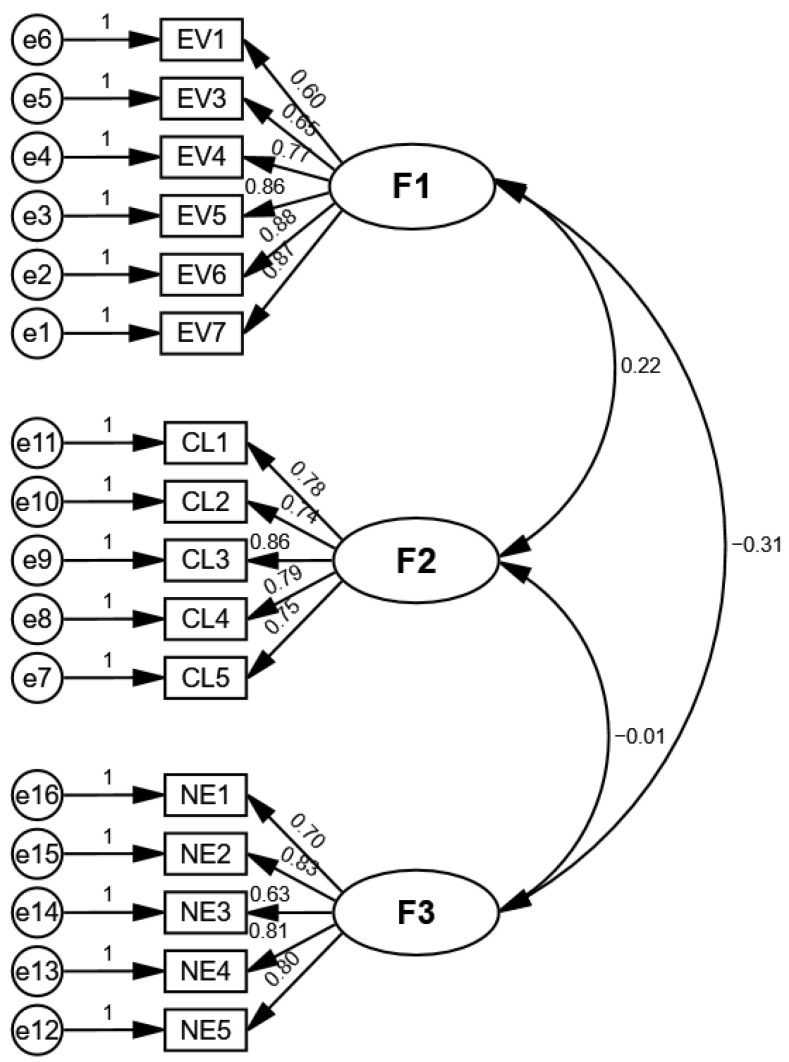
Confirmatory Factor Analysis (CFA) of GACU-T. *Note*. GACU-T = grandparents’ attitudes toward children’s use of technology scale; F1 = Educational Value; F2 = Control and Limitations; F3 = Negative Effects.

Composite reliability (CR) and average variance extracted (AVE) further supported the scale’s construct validity: Educational Value (CR = 0.902, AVE = 0.611), Control and Limitations (CR = 0.888, AVE = 0.615), and Negative Effects (CR = 0.869, AVE = 0.573). Overall, these findings support the reliability and structural validity of the three-factor GACU-T scale (see Figure 1).

To further evaluate discriminant validity, we computed the Heterotrait–Monotrait ratio of correlations (HTMT) among the three GACU-T factors using the full sample. All HTMT values fell below the conventional threshold of 0.85 (EV–CL = 0.194, EV–NE = 0.314, CL–NE = 0.028), providing additional support for discriminant validity beyond the convergent validity evidence reported above.

### 3.2. Latent Profile of Grandparents’ Attitudes

To examine the profiles of grandparents’ attitudes toward young children’s technology use, we conducted latent profile analysis (LPA) across the entire sample using the attitude measures. Table 4 presents the fit indices for the competing models. The bootstrapped likelihood ratio test (BLRT) was statistically significant across all models (*p* < 0.001).

The results indicated that the four-profile model had lower AIC (26,815.720), BIC (27,194.870), and adjusted BIC (26,931.325) values, along with higher entropy (0.90) compared to the two-profile and three-profile solution. Additionally, parsimony and interpretability of the profiles were considered, following best practices for LPA (Lin et al., 2020). Although the five-profile solution showed lower AIC, BIC, and aBIC values and slightly higher entropy than the four-profile solution, the four-profile model was retained based on a comprehensive evaluation of statistical fit, sample composition, theoretical clarity, substantive interpretability, parsimony, and practical relevance. The decision was not based solely on the fact that the smallest class in the five-profile solution accounted for 8.3% of the sample. Rather, the four-profile solution yielded conceptually distinct configurations along the educational-value, control, and risk dimensions, whereas the five-profile solution appeared to split a substantively coherent cluster into two highly similar subgroups without producing a qualitatively new or theoretically meaningful pattern. In addition, the four-profile structure was more consistent with established mediator typologies in the parental and grandparental literature (Nimrod et al., 2022; D. Wu et al., 2020), and the four profiles mapped more clearly onto differentiated and actionable intervention targets for families, communities, and preschools. Classification quality was also comparable across the two solutions, with entropy values of 0.90 for the four-profile model and 0.91 for the five-profile model. Taken together, the four-profile model was selected as the optimal solution because it provided the best balance among statistical fit, theoretical clarity, substantive interpretability, parsimony, and practical relevance.

**Figure 2:**
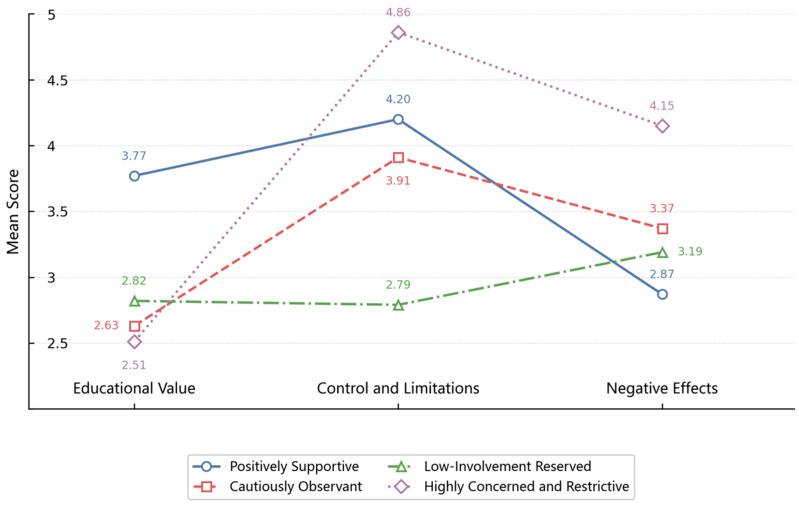
Distribution of the Four Profiles of Grandparents’ Attitudes toward Children’s Technology Use.

As shown in Figure 2 and Table 5, four distinct profiles emerged. The first profile, labeled the “Positively Supportive” group, comprised 43.40% (*n* = 309) of the sample. This group was characterized by the highest score for Educational Value (*M* = 3.77, *SD* = 0.41), higher scores for Control and Limitations (*M* = 4.20, *SD* = 0.47), and the lowest score for Negative Effects (*M* = 2.87, *SD* = 0.73).

The second profile, representing 34.69% (*n* = 247) of the sample, was labeled the “Cautiously Observant” group. This group had lower scores for Educational Value (*M* = 2.63, *SD* = 0.54), and moderate scores for Control and Limitations (*M* = 3.91, *SD* = 0.29), and Negative Effects (*M* = 3.37, *SD* = 0.60).

The third profile, labeled the “Low-Involvement Reserved” group, included 12.50% (*n* = 89) of the sample and had moderate scores for Educational Value (*M* = 2.82, *SD* = 0.67), along with the lowest scores for Control and Limitations (*M* = 2.79, *SD* = 0.51) and moderate scores for Negative Effects (*M* = 3.19, *SD* = 0.80).

The fourth profile, labeled the “Highly Concerned and Restrictive” group, included 9.41% (*n* = 67) of the sample and had the lowest scores for Educational Value (*M* = 2.51, *SD* = 0.63), along with the highest scores for both Control and Limitations (*M* = 4.86, *SD* = 0.23) and Negative Effects (*M* = 4.15, *SD* = 0.75). Descriptive analyses further confirmed significant differences among the four profiles across the three dimensions of grandparents’ attitudes toward children’s technology use (see Table 5).

### 3.3. Effects of Covariates on Grandparental Attitudes Profiles

To address the third research question regarding which children’s and grandparents’ demographic characteristics and caregiving contextual factors predict profile membership, we examined the association between these covariates and the identified profiles of grandparents’ attitudes toward children’s technology use. The three-step LPA approach was used to ensure that the inclusion of covariates did not influence the formation of the latent profiles. Multinomial logistic regression analyses were conducted, with a Bonferroni (1936) correction applied to determine and interpret the significance of the covariates. Initial analyses indicated that four variables were statistically significant: grandparent age (*χ*^2^ = 31.142, *p* < 0.001), education level (*χ*^2^ = 19.190, *p* = 0.024), singleton status (*χ*^2^ = 27.462, *p* < 0.001), and frequency of communication with parents (*χ*^2^ = 29.494, *p* < 0.001). These four variables were subsequently included in the final multinomial logistic regression model. The final model showed a significantly better fit than the intercept-only model (*χ*^2^ = 74.937, *p* < 0.001). The goodness-of-fit indices indicated an acceptable model fit, Pearson (*χ*^2^ = 354.796, *p* = 0.076), and Deviance (*χ*^2^ = 351.224, *p* = 0.097). The likelihood ratio tests further showed that grandparent age (*χ*^2^ = 19.613, *p* = 0.020), singleton status (*χ*^2^ = 19.272, *p* < 0.001) and frequency of communication with parents (*χ*^2^ = 25.169, *p* = 0.003) remained significant predictors of profile membership (see Table 6). Education level was not retained as a significant predictor in the final model.

The “Positively Supportive” group (Profile 1) was used as the reference category because it included the largest number of grandparents. Therefore, the odds ratios reported in Table 7 indicate the odds of belonging to Profiles 2, 3, or 4 relative to Profile 1. For the categorical predictors, the reference categories were grandparents aged 66 years and older, grandchildren with sibling(s), and those who never communicated with parents about childcare. As shown in Table 7, several key findings emerged. First, compared with grandparents aged 66 years and older, those aged 61–65 years had higher odds of belonging to the “Low-Involvement Reserved” group rather than the Positively Supportive” group (OR = 2.105, *p* = 0.039), and to the “Highly Concerned and Restrictive” group rather than the “Positively Supportive” group (OR = 2.674, *p* = 0.034). Second, expressed using inverse odds ratios, grandparents of only children were 1.60 times more likely to belong to the “Positively Supportive” group rather than the “Cautiously Observant” group, and 3.02 times more likely to belong to the “Positively Supportive” group rather than the “Low-Involvement Reserved” group, compared with grandparents whose grandchildren had sibling(s). Third, expressed inversely, grandparents who never communicated with parents about childcare had 3.08 times higher odds of belonging to the “Cautiously Observant” group rather than the “Positively Supportive” group, and 2.99 times higher odds of belonging to the “Low-Involvement Reserved” group rather than the “Positively Supportive” group, compared with those who communicated more frequently.

## 4. Discussion

This study examined grandparents’ attitudes toward their grandchildren’s technology use in Shanghai, China. Latent profile analysis identified four distinct attitudinal patterns—Positively Supportive, Cautiously Observant, Low-Involvement Reserved, and Highly Concerned and Restrictive—with the largest proportion of grandparents classified as Positively Supportive. The findings further indicated that grandparents’ age, sibling status, and frequency of communication between grandparents and parents were significant predictors of profile membership.

### 4.1. Reliability and Validity of the GACU-T Scale

The GACU-T scale, grounded in a three-construct framework—educational value, control and limitations, and negative effects—was designed to capture grandparents’ perceptions of the benefits, risks, and mediation of children’s technology use (Türel & Gür, 2019). The findings support the scale’s construct validity in the Chinese context. The three constructs were interrelated; for example, both educational value and negative effects were associated with control and limitations, reflecting the integrated nature of grandparents’ evaluative and regulatory perspectives.

The scale items addressed key dimensions of digital caregiving, including perceived educational benefits, mediation roles, and potential physical and psychological impacts on children. Together, these dimensions provide a comprehensive representation of grandparental attitudes. The GACU-T demonstrated satisfactory reliability and convergent validity, supporting its use as a measurement tool in future research on digital grandparenting.

### 4.2. Differentiated and Ambivalent Attitudes Toward Children’s Technology Usage

Overall, grandparents’ attitudes toward children’s technology use were moderate (*M* = 3.42), suggesting a more cautious stance compared to findings from studies of younger parents, who tend to report more favorable attitudes (K. V. Tan & Zhooriyati, 2021; D. Wu et al., 2020). One contributing factor may be differences in digital competence. Prior work has shown that caregivers’ digital competence is associated with their attitudes and mediation strategies (Nathanson, 2015; Nikken & Opree, 2018; Nikken & Schols, 2015). Many grandparents have more limited experience with digital technologies (Cheng et al., 2018), which may constrain their confidence in guiding children’s digital engagement (N. Hu & Ning, 2020).

The four identified profiles highlight substantial heterogeneity in grandparents’ attitudes. The Positively Supportive group (43.40%) combined strong recognition of educational value with relatively high levels of regulation and low perceived risk. This pattern suggests a conditional acceptance of technology—valued when closely supervised and supported by clear boundaries. Such a profile is understandable in the Chinese sociocultural context, where educational achievement is highly valued and digital tools may be accepted when they are seen as serving children’s learning and development (Jiang & Monk, 2016). This pattern resonates with Nimrod et al.’s (2022) highly involved mediators and with D. Wu et al.’s (2020) high-ICT-proficiency parental profile, suggesting that grandparents in this group occupy a relatively high-digital literacy position within the broader intergenerational landscape and may also draw on shared family leisure routines that support co-use (Hebblethwaite, 2016).

The Cautiously Observant group (34.69%) reflected lower perceived educational value alongside moderate regulation and perceived risk, indicating a more tentative stance. The Low-Involvement Reserved profile (12.50%) was marked by relatively low educational value, the lowest level of control and limitations, and moderate perceived negative effects. Compared with the Cautiously Observant group, this profile appears to reflect a more passive or detached response, in which grandparents remain unconvinced of technology’s educational value but are also less inclined to intervene actively. In contrast, the Highly Concerned and Restrictive profile (9.41%) was characterized by the lowest perceived educational value and the highest levels of both control and perceived negative effects, suggesting a strongly risk-averse perspective accompanied by restrictive mediation. Read through TAM, the Cautiously Observant pattern reflects low perceived usefulness combined with moderate perceived risk, broadly consistent with Jiang and Monk’s (2016) account of academically focused caution in Chinese families. The Low-Involvement Reserved profile is more naturally interpreted through intergenerational digital-divide theory, in which limited digital literacy can manifest as both low perceived usefulness and disengagement (Cheng et al., 2018; Friemel, 2016). The Highly Concerned and Restrictive profile parallels risk-dominant discourses in pediatric screen-time guidance (Domingues-Montanari, 2017; Hill et al., 2016).

Taken together, these profiles suggest that grandparents’ attitudes are not easily captured along a simple positive–negative continuum. Rather, they reflect varying combinations of perceived usefulness, perceived risk, and regulatory inclination. From the perspective of the Technology Acceptance Model, grandparents in the Positively Supportive group may perceive technology as both useful and manageable, whereas those in the Highly Concerned and Restrictive group appear to emphasize risk and therefore adopt stronger control strategies (Davis, 1989; Granić & Marangunić, 2019). The two intermediate profiles further suggest that some grandparents may remain uncertain or insufficiently confident in responding to the challenges of digital caregiving. Overall, these findings underscore the ambivalent and differentiated nature of grandparents’ attitudes and suggest that many grandparents are still in the process of negotiating how to support, regulate, or distance themselves from children’s technology use.

### 4.3. Key Factors Shaping Grandparents’ Attitudes

Multinomial logistic regression analyses identified several factors associated with profile membership. Grandparent age was a significant predictor. Compared with those aged 66 and older, grandparents aged 61–65 were more likely to belong to the Low-Involvement Reserved and Highly Concerned and Restrictive profiles. This pattern may reflect a group that is actively engaged in caregiving but navigating technology with varying levels of confidence.

Family structure also played a role. Grandparents of children with siblings were more likely to fall into less involved or less restrictive profiles. This aligns with prior findings suggesting that caring for multiple children may reduce the time and attention available for monitoring technology use (Gou & Dezuanni, 2018; Nimrod et al., 2022).

Caregiving context, particularly communication between grandparents and parents, was also a significant predictor. Grandparents who communicated more frequently with parents tended to adopt more engaged and balanced approaches, whereas those with limited communication were more likely to fall into less involved profiles. These findings suggest that intergenerational communication may support more informed and confident approaches to digital caregiving. Prior research has linked such exchanges to reductions in the digital divide among older adults (Friemel, 2016), which may, in turn, shape attitudes toward children’s technology use.

### 4.4. Contributions, Implications and Limitations

The present study makes three sets of contributions. Theoretically, it extends digital-caregiving research from parents to grandparents, integrates TAM with intergenerational digital-divide theory, and offers an empirically grounded typology that captures configuration rather than general patterns of attitudes. Although grandparents play a substantial daytime caregiving role for many Chinese preschool-age children, their perspectives have been comparatively under-examined in the digital-caregiving literature (Bates & Taylor, 2013; Dunifon et al., 2018), and the present study contributes evidence on this group.

Methodologically, the study adapts and validates a grandparent-specific instrument (GACU-T) and applies a three-step latent profile analysis to identify and predict latent profiles of attitudes. Practically, the four profiles yielded conceptually clearer and more interpretable patterns. Families, community organizations, and preschools can tailor digital literacy supports and parent–grandparent communication scaffolds to each profile, rather than treating grandparents as an undifferentiated group.

These contributions have several implications for intergenerational digital support. Consistent with research on digitally transitional populations, the identified profiles suggest that differences in grandparents’ digital confidence, literacy, and perceptions of technology may shape how they engage with children’s digital experiences. Accordingly, family members, preschools, and community organizations may benefit from providing differentiated support for grandparents, including opportunities for intergenerational learning, communication about digital parenting practices, and guidance regarding balanced and developmentally appropriate technology use. Public policies promoting digital inclusion among older adults should also recognize grandparents in their caregiving role rather than viewing them solely as end users of digital services.

In addition, preschools and kindergartens can play a role in designing and implementing programs that engage grandparents and support the development of attitudes informed by an evidence base. Such efforts may include parent meetings, family education initiatives, and interactive home–school communication platforms.

Finally, young parents play a crucial role as close partners in parenting, influencing grandparents’ attitudes toward technology. It is essential for young parents to regularly communicate and share their digital parenting experiences with grandparents, helping them understand and evaluate technology thoughtfully and adopt effective mediation strategies for managing children’s technology use.

Several limitations should also be acknowledged. First, all participants were recruited from Shanghai, one of the most economically developed regions in China, which may limit the generalizability of the findings to other geographical and socioeconomic contexts. In addition, participants were recruited through a grandparenting seminar, which may have introduced self-selection bias. Future studies should therefore include more geographically and socioeconomically diverse samples.

Second, the study relied on grandparents’ self-reports, which may not fully reflect actual attitudes or caregiving practices. Future research could incorporate multi-source or observational data to provide a more comprehensive understanding of grandparental digital caregiving.

Third, the cross-sectional design limits the ability to examine changes in attitudes over time or draw causal inferences regarding the relationships among demographic, caregiving, and attitudinal factors. Longitudinal and mixed-methods studies would help clarify how grandparents’ attitudes evolve and how parent–grandparent interactions shape digital caregiving practices over time.

## Figures and Tables

**Table 1:** Demographic characteristics for participants (*n* = 712).

Variables	Number	Percent (%)
Grandparent identity		
Paternal grandfather	144	20.23
Paternal grandmother	339	47.61
Maternal grandfather	44	6.18
Maternal grandmother	185	25.98
Grandparent age		
55 years and younger	212	29.78
56–60 years	227	31.88
61–65 years	152	21.35
66 years and older	121	16.99
Grandparent highest education		
Primary or below	196	27.53
Junior secondary	322	45.22
High school	133	18.68
University/college degree and above	61	8.57
Grandparent employment status		
Part-time	39	5.48
Full-time	121	16.99
Retiree/unemployed	552	77.53
Grandchild age		
0–35 months old	32	4.49
36–72 months old	680	95.51
Grandchild gender		
Boy	393	55.20
Girl	319	44.80
Singleton		
Only child	359	50.42
Sibling(s)	353	49.58
Co-habitation with grandchildren		
Yes	453	63.62
No	259	36.38
Communication with parents		
Frequently	122	17.14
Occasionally	359	50.42
Rarely	163	22.89
Never	68	9.55

**Table 2:** Exploratory factor analysis results of GACU-T.

Item	Factor Loading			Commonality
	Educational Value	Control and Limitations	Negative Effects	
EV1	0.724			0.538
EV3	0.800			0.647
EV4	0.815			0.686
EV5	0.857			0.764
EV6	0.887			0.817
EV7	0.855			0.770
CL1		0.836		0.722
CL2		0.832		0.706
CL3		0.881		0.781
CL4		0.852		0.729
CL5		0.824		0.688
NE1			0.797	0.648
NE2			0.872	0.777
NE3			0.725	0.542
NE4			0.861	0.755
NE5			0.822	0.701
Variance contribution of each factor (%)	31.827	22.234	16.393	

Note. The extraction method: Principal component analysis method. Rotation method: Kaiser normalized maximum variance method; The rotation has converged after 5 iterations; GACU-T = Grandparents’ attitudes toward children’s technology use scale; EV = Educational Value; CL = Control and Limitations; NE = Negative Effects.

**Table 3:** Results of Reliability Analysis of GACU-T.

GACU-T Scale	Number of Items	Cronbach’s Alpha (α)
Educational Value	6	0.908
Control and Limitations	5	0.903
Negative Effects	5	0.880
Overall scale	16	0.754

Note. GACU-T = Grandparents’ attitudes toward children’s technology use scale.

**Table 4:** Model fit statistics for determining the optional number of profiles.

Profile	AIC	BIC	aBIC	Entropy	LMRT (*p*)	BLRT (*p*)	Class Size Per Profile
1	29,839.850	29,986.030	29,884.421	1	-	-	-
2	28,126.390	28,350.230	28,194.641	0.85	<0.001	0.01	48.6%, 51.4%
3	27,242.540	27,544.040	27,334.470	0.88	<0.001	0.01	55.6%, 26.1%, 18.3%
4	26,815.720	27,194.870	26,931.325	0.90	<0.001	0.01	43.4%, 34.7%, 12.5%, 9.4%
5	26,097.010	26,553.820	26,236.294	0.91	<0.001	0.01	11.4%, 34.0%, 34.8%, 11.5%, 8.3%

Note. AIC = Akaike information criteria; BIC = Bayesian information criteria; aBIC = Sample-size adjusted Bayesian information criteria; LMRT = Lo–Mendel–Rubin likelihood ratio test; BLRT = Bootstrapped likelihood ratio test.

**Table 5:** Means of Grandparents’ Attitudes toward Children’s Technology Use Across Four Profiles.

Profile	*n*	Percent	Educational Value (*M* ± *SD*)	Control and Limitations (*M* ± *SD*)	Negative Effects (*M* ± *SD*)
Positively Supportive	309	43.40%	3.77 ± 0.41	4.20 ± 0.47	2.87 ± 0.73
Cautiously Observant	247	34.69%	2.63 ± 0.54	3.91 ± 0.29	3.37 ± 0.60
Low-Involvement Reserved	89	12.50%	2.82 ± 0.67	2.79 ± 0.51	3.19 ± 0.80
Highly Concerned and Restrictive	67	9.41%	2.51 ± 0.63	4.86 ± 0.23	4.15 ± 0.75

Note: *M* = Mean; *SD* = Standard deviation.

**Table 6:** Likelihood ratio tests of the multinomial logistic regression model.

Model Analysis	*χ* ^2^	*df*	*p*
Model likelihood ratio test	74.937	21	<0.001
Goodness-of-fit: Pearson	354.796	318	0.076
Goodness-of-fit: Deviance	351.224	318	0.097
Parameter likelihood ratio tests			
Grandparent age	19.613	9	0.020
Singleton status	19.272	3	<0.001
Communication with parents	25.169	9	0.003

Note: *df* = Degree of freedom.

**Table 7:** Multinomial logistic regression model results.

Profile ^a^		B	*SE*	Wald	*df*	*p*	OR
Profile 2	Intercept	0.396	0.344	1.328	1	0.249	
	Grandparent age: 55 years and younger	0.063	0.274	0.053	1	0.818	1.065
	Grandparent age: 56–60 years	−0.049	0.267	0.034	1	0.854	0.952
	Grandparent age: 61–65 years	0.082	0.297	0.076	1	0.783	1.085
	Grandparent age: 66 years and older	0 ^b^			0		
	Singleton: Only child	−0.469	0.182	6.639	1	0.010	0.626
	Singleton: Sibling(s)	0 ^b^			0		
	Communicated frequently with parents	−1.123	0.376	8.902	1	0.003	0.325
	Communicated occasionally with parents	−0.400	0.322	1.545	1	0.214	0.670
	Communicated rarely with parents	0.003	0.347	0.000	1	0.993	1.003
	Never communicated with parents	0 ^b^			0		
Profile 3	Intercept	−0.151	0.420	0.130	1	0.718	
	Grandparent age: 55 years and younger	−0.167	0.392	0.181	1	0.671	0.847
	Grandparent age: 56–60 years	−0.481	0.392	1.506	1	0.220	0.618
	Grandparent age: 61–65 years	0.745	0.361	4.250	1	0.039	2.105
	Grandparent age: 66 years and older	0 ^b^			0		
	Singleton: Only child	−1.106	0.274	16.265	1	0.000	0.331
	Singleton: Sibling(s)	0 ^b^			0		
	Communicated frequently with parents	−1.098	0.475	5.340	1	0.021	0.334
	Communicated occasionally with parents	−0.839	0.410	4.196	1	0.041	0.432
	Communicated rarely with parents	−0.159	0.431	0.136	1	0.713	0.853
	Never communicated with parents	0 ^b^			0		
Profile 4	Intercept	−1.345	0.553	5.917	1	0.015	
	Grandparent age: 55 years and younger	0.201	0.485	0.171	1	0.679	1.222
	Grandparent age: 56–60 years	0.372	0.460	0.654	1	0.419	1.450
	Grandparent age: 61–65 years	0.984	0.465	4.481	1	0.034	2.674
	Grandparent age: 66 years and older	0 ^b^			0		
	Singleton: Only child	−0.472	0.283	2.774	1	0.096	0.624
	Singleton: Sibling(s)	0 ^b^			0		
	Communicated frequently with parents	−0.280	0.524	0.286	1	0.593	0.756
	Communicated occasionally with parents	−0.399	0.485	0.676	1	0.411	0.671
	Communicated rarely with parents	−0.456	0.552	0.683	1	0.408	0.634
	Never communicated with parents	0 ^b^			0		

Note. The reference outcome category was Profile 1. Thus, the odds ratios for Profiles 2, 3, and 4 indicate the odds of belonging to each comparison profile relative to Profile 1. ^a^ = The reference category is: profile 1, ^b^ = This parameter is set to zero because it is redundant, B = B-coefficient; *SE* = Standard error; Wald = Wald chi-square test; *df* = Degree of freedom; OR = Odds ratio; Profile 1 = Positively Supportive; Profile 2 = Cautiously Observant; Profile 3 = Low-Involvement Reserved; Profile 4 = Highly Concerned and Restrictive.

## Data Availability

The data presented in this study are available on reasonable request from the corresponding author. The data are not publicly available due to privacy and ethical restrictions.
